# Study on the Formation and Separation Process of Droplets in the Medical Piezoelectric Atomization Device Induced by Intra-hole Fluctuation

**DOI:** 10.1186/s10033-022-00754-9

**Published:** 2022-06-11

**Authors:** Qiufeng Yan, Wanting Sun, Jianhui Zhang

**Affiliations:** 1 School of Electrical Engineering, Nantong University, Nantong, 226019 China; 2 School of Materials Science and Engineering, Harbin Institute of Technology, Harbin, 150001 China; 3 College of Mechanical and Electrical Engineering, Guangzhou University, Guangzhou, 510006 China

**Keywords:** CLSM, Intra-hole fluctuation, Piezoelectric atomization, Droplet distribution

## Abstract

Traditional atomization devices always exhibit many drawbacks, such as non-uniform atomization particle sizes, instability of transient atomization quantity and uncontrollability of precise energy, which seriously restrict further practical application of atomization inhalation therapy. The formation and separation process of droplets belongs to a microphenomenon of atomization. The investigation of the droplet formation and separation process will be favorable for understanding the atomization mechanism. In present work, the Conservative Level Set Method (CLSM) is successfully applied on the simulation of the formation and separation of droplets in a medical piezoelectric atomization device induced by intra-hole fluctuation. The intra-hole fluctuation mechanism is systematically explored and analyzed, and also the expression of the volume change in the micro cone hole is built and evaluated. Both the control equation and simulation model of droplet formation and separation process have been well established by meshing the simulation model, and thereby the process of droplet formation and separation is simulated. The corresponding results demonstrate that the breaking time of droplets is decreased with the inlet velocity and liquid temperature rising, while enhanced with the liquid concentration increasing. Meanwhile, the volume of droplet is decreased with the inlet velocity and liquid concentration increasing, but increased with the liquid temperature rising. The velocity of droplet is enhanced with the inlet velocity and liquid temperature rising, and reduced with the increase of liquid concentration. When the large side diameter of micro-cone hole is set as 79 μm, the breaking time of the droplet reaches a minimum value of 38.7 μs, whereas the volume and the velocity of droplet reach a maximum value of 79.8 pL and 4.46 m/s, respectively. This study provides theoretical guidance for the design of medical piezoelectric atomization devices and contributes to the promotion of inhalation therapy in practical use.

## Introduction

The corona virus disease 2019 (COVID-19) has swept the whole world since the end of 2019, thus it caused serious economic losses and casualties [1–3]. Up to now, there is still lacking specific medicine and vaccines precaution for COVID-19 virus. During the progress of fighting with the virus, the autoimmune ability of patients is particularly important. Interferon-*β*, as one of the anti-virus treatment approaches, is inhaled by atomization to enhance the autoimmune function by stimulating human immune cells [4]. Interferon-*β* can be fully dispersed into tiny droplets produced by the effect of atomization device, which directly acts on respiratory epithelial cells. It is noteworthy that the inhalation therapy contains a relatively small dosage, avoiding and/or reducing the systemic medication so as to obviously weaken the toxic and side effects of the drugs. Simultaneously, inhalation therapy can relive the pain deriving from the injection and administration, since its operation is simple and convenient [5]. Accordingly, due to these advantages mentioned above, many scholars have focused on the investigation of the medical atomization device to satisfy the needs of atomization therapy.

In 2006, Vecellio [6] designed a kind of medical atomizer, which adopt electroplating technology to process 6000 3-µm-diameter holes on the mesh plate. Under the excitation of the alternating current voltage, the piezoelectric transducer generates high-frequency vibrations to cause the atomization of the drug solution to form an aerosol. The respiratory system is directly connected with the lesion, achieving targeted and quantitative administration and reducing the side effects caused by systemic administration. In 2011, the laser ablation technology and electroforming technology were utilized by Lin et al. to prepare a palladium-nickel alloy nozzle plate having a nozzle diameter of 5 μm [7]. The nozzle plate prepared using such method is quite difficult and can be used at a frequency of 100 kHz. Studies have shown that the nozzle can effectively control the particle size of drug droplets during the progress of atomizing a drug solution. The average particle size of the atomized droplets is approximately 3 μm, which satisfies the requirement that the drug particles must be less than 4 μm when absorbed by the lungs. In 2012, Beck-Broichsitter et al. [8] proposed a vibrating-mesh-type nebulizer to atomize the prepared nanoparticle drugs. Experiments have confirmed that using nanoparticles to encapsulate sildenafil can maintain its stability and that atomizing the process does not affect the particle size, particle size distribution, or sildenafil content. In addition, other works on the inhalation therapy are also documented [9–11].

Both the atomization rate and particle size are expected to play a pronounced role in the inhalation therapy. Olseni et al. [12, 13] found that when the average size of drug particle was smaller than 5 μm, the drugs can be effectively delivered to the airway and lungs, and thereby deposited on a lesion by gravity.

It should be pointed out that for drug particles deposited on the lesion, the mean diameter of drug particles cannot be larger than 4 μm. Simultaneously, a constant atomization rate can make the patients feel more comfortable during the treatment process.

In the process of atomization, the formation and separation of droplets are considered as a typical gas-liquid two-phase flow. It is difficult in tracing the free interface of gas-liquid two-phase flow. A Marker-And-Cell (MAC) method [14] to track the position of free surface was developed by Harlow et al. Subsequently, scholars proposed some methods to solve these problems. For instance, Simplified-MAC (SMAC) [15], Volume of Fluid (VOF) [16], Level Set Method (LSM) [17].

Currently, VOF and LSM are regarded as two most commonly free interface tracking approaches. By comparison, LSM seems to be more popular in interface tracking application, because the information of unit normal vector and curvature radius of interface can be obtained. This overcomes the shortcomings of VOF method deriving from the difficulties in simulating the necessary unit normal vector and curvature radius [18]. Nevertheless, the traditional LSM also shows poor conservation easily resulting in mass loss, thus affecting the accurate positioning of the interface leads to an appearance of wrong results. In order to improve the conservation of mass, in the convective phase of the level set, several hybrid methods have been employed, for instance, combining LSM with VOF to obtain VOF-LSM [19]. However, this algorithm will increase the complexity of computation and waste the advantages of LSM. For simplification of the calculation process of VOF-LSM and promotion in the accuracy of LSM, Olsson et al. [20] proposed a CLSM approach on the basis of LSM. The key point of CLSM is to utilize a 0.5 horizontal plane working as the gas-liquid interface, which can successfully improve the calculation accuracy [21–24].

The formation and separation process of droplets is ascribed to be a micro phenomenon of atomization. Studies on droplet formation and separation process will be conducive to understand the atomization mechanism. In this study, the CLSM is successfully adopted on the simulation of the formation and separation of droplets in a medical piezoelectric atomization device induced by intra-hole fluctuation. The intra-hole fluctuation mechanism is systematically analyzed, and the expression of the volume change of the micro cone hole is evaluated. Both the control equation and simulation model of droplet formation and separation process are established. Simultaneously, the process of droplet formation and separation is simulated. This study reveals the atomization mechanism of the medical piezoelectric atomization device induced by intra-hole fluctuations from a micro perspective. It is anticipated to provide some theoretical guidance for the design of medical piezoelectric atomization devices and contribute to the promotion of inhalation therapy in practical use.

## Construction of Atomization Experiment Platform

**Figure 1:**
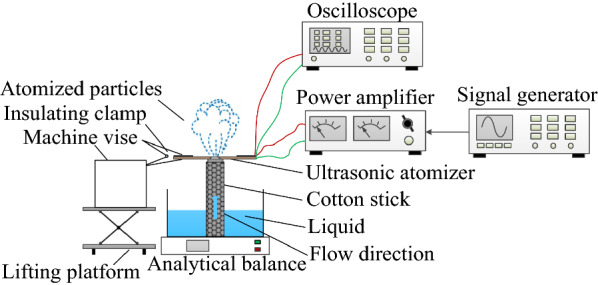
Atomization experiment platform of the medical piezoelectric atomization device induced by intra-hole fluctuations

**Figure 2:**
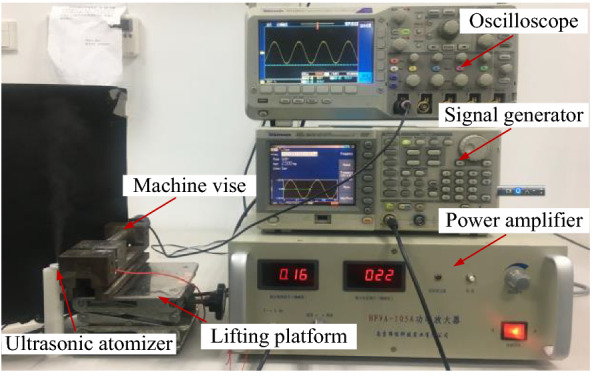
Photograph of the atomization experiment of the medical piezoelectric atomization device induced by intra-hole fluctuations

Figure 1 shows the atomization experiment platform of the medical piezoelectric atomization device induced by intra-hole fluctuation. Insulating clamps were used to mount the atomizer piece and fixed by a machine vise, which was placed on the lifting platform. The height of the atomizer piece was adjusted by the lifting platform to make sure the atomizer piece only touch the cotton stick (there was no gap or applied force between the sheet atomizer piece only touched the cotton stick (there was no gap or applied force between the sheet and the swab). The cotton stick was placed in a container filled with liquid. Because of the capillary force action, the liquid was flowed from the container to the atomizer piece as indicated by the blue arrow. Under the excitation of AC signal, the inner wall of the micro cone hole will fluctuate and cause the change of the volume of the micro cone hole. Simultaneously, because of the existence of the difference between the positive and negative flow resistance of the micro cone hole, it will produce a pumping effect [25–30] to remove the liquid from the cotton stick to the external environment, thereby producing the atomization phenomenon. Figure 2 illustrates the photograph of the atomization experiment of the medical piezoelectric atomization device induced by intra-hole fluctuation.

**Figure 3:**
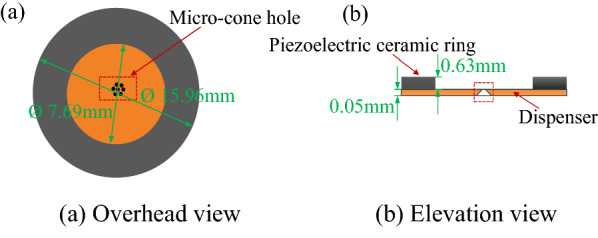
The schematic diagram showing the structure and parameters of the atomizer piece used in this work

Figure 3 shows the structure and parameters of the atomizer piece. It can be seen from Figure 3 that the piezoelectric vibrator is composed of dispenser and piezoelectric ceramic ring. The outer diameter, inner diameter, and thickness of the piezoelectric ceramic ring are set as values of 15.96 mm, 7.69 mm and 0.63 mm, respectively, while the diameter and thickness of the dispenser are 15.96 mm and 0.05 mm respectively. The taper holes with diameter of 740 μm were machined in the center area of the piezoelectric atomizer.

## Theoretical Analysis of the Intra-hole Fluctuation Mechanism in the Medical Piezoelectric Atomization Device

**Figure 4:**
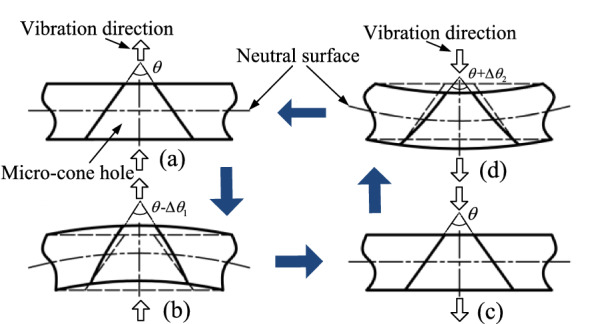
The schematic diagram of deformation process of micro-cone hole in a cycle

Figure 4 shows the deformation process of micro-cone hole in a cycle. Under the excitation of AC voltage, the piezoelectric ceramic ring can generate alternating compressive and tensile forces on the dispenser to drive the vibration of the dispenser. As the micro cone hole is located on the substrate, the inner wall of the micro-cone hole can fluctuate with the vibration of the dispenser causing a change in volume, and then the pressure change inside the micro-cone hole can produce atomization. This cycle continues as long as the piezoelectric ceramic ring is remained to deliver the required stimulation.

**Figure 5:**
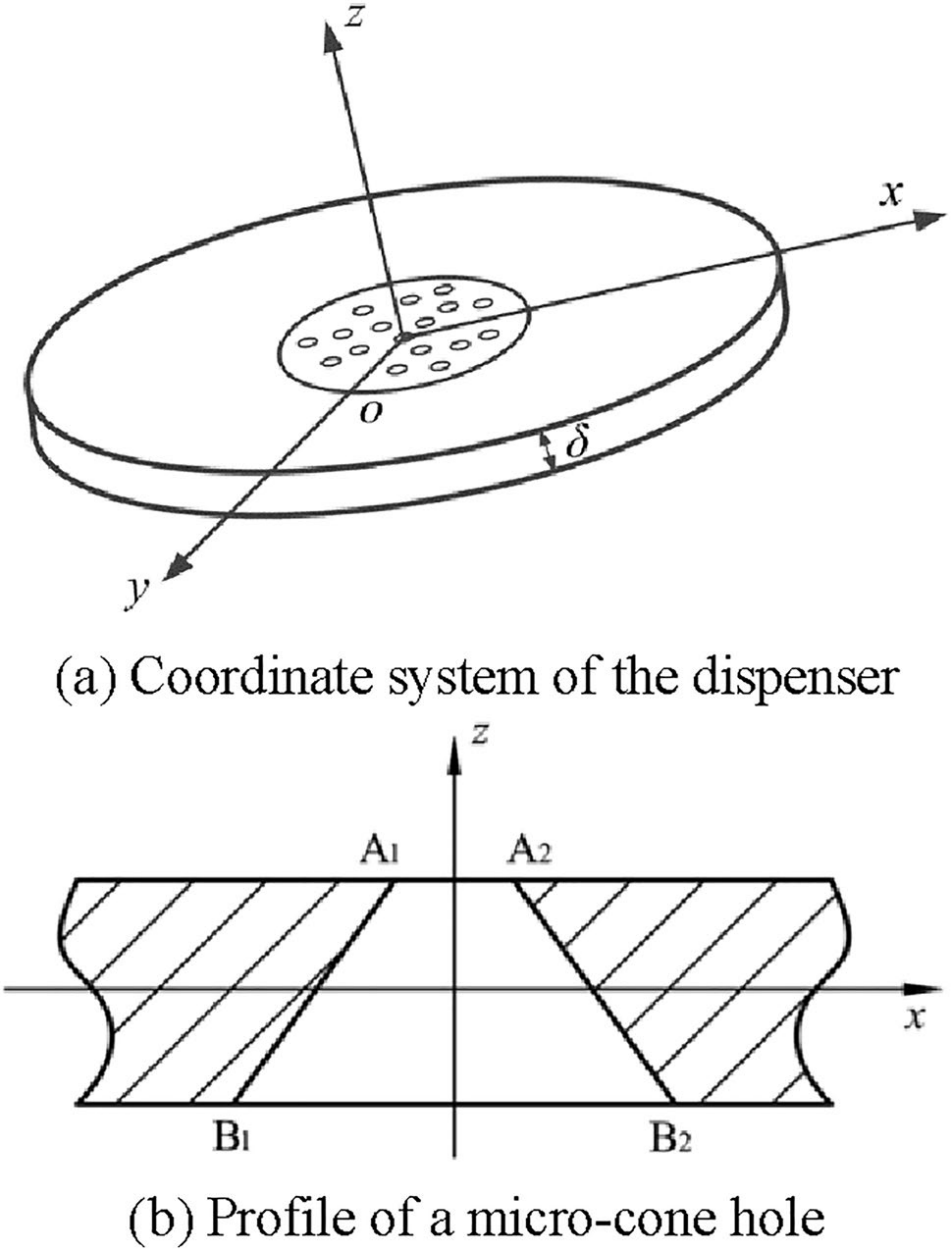
Schematic diagram of coordinate settings of the dispenser [25]

The coordinate system is built by Kirchhoff hypothesis, the neutral surface of the dispenser is set as the *xy* plane and the *z* axial is perpendicular to it, as shown in Figure 5(a). According to the Kirchhoff hypothesis, the velocity and amplitude of the upper and lower vibrations of the dispenser are symmetric with respect to the equilibrium position. In order to better illustrate the deformation of the apertures, the *xz* plane is shown in Figure 5(b). Simultaneously, there is no movement of the dispenser neutral surface in *x* and *y* directions. Any straight line which is vertical to the neutral surface before its deformation is still vertical to the elastic and flexible plane after its deformation. The length of the line remains the same during this progress.

**Figure 6:**
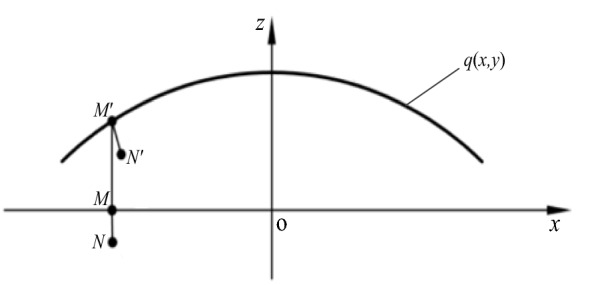
Deformation process of a point in the dispenser

When the neutral surface is set as a plane of *q*(*x*,*y*), the movement from *N* to *N*' on the dispenser is given in Figure 6, in which the *MN* line is perpendicular to the *xy* plane, and *M'N'* line is perpendicular to the surface *q*(*x*,*y*).

After the occurrence of deformation, the point of *M*(*x*_0_, *y*_0_, 0) is changed to another position as *M*' [*x*_0_, *y*_0_, *q*(*x*_0_, *y*_0_)]. According to Kirchhoff hypothesis, Eq. (1) is the normal format at *M*' on the neutral surface after the deformation:

1 $$\left\{ {\begin{array}{*{20}l} {x = x_{0} - q_{x} \left[ {z - q(x_{0} ,y_{0} )} \right],} \\ {y = y_{0} - q_{y} \left[ {z - q(x_{0} ,y_{0} )} \right],} \\ \end{array} } \right.$$

where *q*_*x*_ and *q*_*y*_ refer to the partial derivatives of *q*(*x*,*y*) at *x*_0_ and *y*_0_, respectively.

According to Kirchhoff hypothesis, after calculation the equation of $\left| {MN} \right| = \left| {M^{\prime}N^{\prime}} \right| = \left| {z_{0} } \right|$ can be achieved.

2 $$\left[ {(x_{0} - x_{0}^{^{\prime}} )^{2} + (y_{0} - y_{0}^{^{\prime}} )^{2} + (q(x_{0} ,y_{0} ) - z_{0}^{^{\prime}} )^{2} } \right]^{\frac{1}{2}} = \left| {z_{0} } \right|.$$

The coordinate of *N'* is calculated by Eqs. (1) and (2), and the result is shown as Eq. (3).

3 $$(x_{0} - bq_{x} ,y_{0} - bq_{y} ,b + q)$$

where $$b = {\raise0.7ex\hbox{${z_{0} }$} \!\mathord{\left/ {\vphantom {{z_{0} } {\left( {1 + q_{x}^{2} + q_{y}^{2} } \right)^{\frac{1}{2}} }}}\right.\kern-\nulldelimiterspace} \!\lower0.7ex\hbox{${\left( {1 + q_{x}^{2} + q_{y}^{2} } \right)^{\frac{1}{2}} }$}}$$.

The surface equation for a micro-cone hole is written by Eq. (4).

4 $$\left( {x^{2} + y^{2} } \right)^{\frac{1}{2}} = - \tan \left( {\frac{\alpha }{2}} \right) \cdot z + r_{m} ,$$

where $\alpha$ is the half angle of micro-cone hole, and *r*_*m*_ is the diameter of the circle on its neutral surface.

In order to estimate the entire volume changes of the micro-cone hole, it’s necessary to calculate the volume changes of its micro unit.

It is assumed that a micro unit at point *E*_1_(*x*, *y*, *z*), its dimensions are Δ*x*, Δ*y* and Δ*z*, respectively. The result of its volume before the deformation is calculated by Eq. (5).

5 $$\Delta V_{{\text{f}}} = \Delta x \cdot \Delta y \cdot \Delta z.$$

With the help of second order Taylor expansion of the function at point *E*_1_(*x*, *y*, *z*), and the ignorance of the infinitesimal of high order, the vectors of the three sides of the micro unit at the top after deformation can be obtained using the Eqs. (6)–(8).

6 $${\varvec{v}}_{x} = \left[ \begin{gathered} \Delta x - bq_{xx} \Delta x - b_{x} q_{x} \Delta x, \hfill \\ - \left( {bq_{yx} \Delta x + b_{x} q_{y} \Delta x} \right), \hfill \\ b_{x} \Delta x + q_{x} \Delta x \hfill \\ \end{gathered} \right],$$

7 $${\varvec{v}}_{y} = \left[ \begin{gathered} - \left( {bq_{xy} \Delta y + b_{y} q_{x} \Delta y} \right), \hfill \\ \Delta y - bq_{yy} \Delta y - b_{y} q_{y} \Delta y, \hfill \\ b_{y} \Delta y + q_{y} \Delta y \hfill \\ \end{gathered} \right],$$

8 $${\varvec{v}}_{z} = \left[ { - b_{z} q_{x} \Delta z, - b_{z} q_{y} \Delta z,b_{z} \Delta z} \right].$$

As mentioned above, the dispenser is vibrating symmetrically to the original neutral surface. For simplified the calculation, it is reasonable to assume that the function of the neutral surface is *q*(*x*, *y*) when the dispenser reaches the highest position during vibrations, and the function of the neutral surface is -*q*(*x*, *y*) when the dispenser reaches the lowest position during vibrations.

The volumes of a micro-cone hole can be evaluated by Eq. (9) when the dispenser reaches the highest positions during the vibration progress.

9 $$V_{u} = \iiint\limits_{\Omega } {\left[ \begin{gathered} s^{\frac{1}{2}} + z^{2} \left( {q_{xx} q_{yy} - q_{xy}^{2} } \right)s^{{ - \frac{3}{2}}} + \hfill \\ z\left( \begin{gathered} - q_{yy} - q_{xx} - q_{y}^{2} q_{xx} + \hfill \\ 2q_{x} q_{y} q_{xy} - q_{x}^{2} q_{yy} \hfill \\ \end{gathered} \right)s^{ - 1} \hfill \\ \end{gathered} \right]}{\text{d}}V,$$

where $$s=1+{q}_{x}^{2}+{q}_{y}^{2}$$.

The volumes of a micro-cone hole can be calculated by Eq. (10) when the dispenser reaches the lowest positions during vibrations.

10 $$V_{d} = \iiint\limits_{\Omega } {\left[ \begin{gathered} s^{\frac{1}{2}} + z^{2} \left( {q_{xx} q_{yy} - q_{xy}^{2} } \right)s^{{ - \frac{3}{2}}} + \hfill \\ z\left( \begin{gathered} q_{yy} + q_{xx} + q_{y}^{2} q_{xx} - \hfill \\ 2q_{x} q_{y} q_{xy} + q_{x}^{2} q_{yy} \hfill \\ \end{gathered} \right)s^{ - 1} \hfill \\ \end{gathered} \right]}{\text{d}}V,$$

where $$s=1+{q}_{x}^{2}+{q}_{y}^{2}$$.

The maximum change (Eq. (11)) of the micro-cone hole volume during vibrations can be confirmed by the subtraction of Eqs. (9) and (10).

11 $$\Delta V_{K} = \iiint\limits_{\Omega } {\left[ {2z\left( \begin{gathered} q_{yy} + q_{xx} + q_{y}^{2} q_{xx} - \hfill \\ 2q_{x} q_{y} q_{xy} + q_{x}^{2} q_{yy} \hfill \\ \end{gathered} \right)s^{ - 1} } \right]}{\text{d}}V,$$

where $$s=1+{q}_{x}^{2}+{q}_{y}^{2}$$.

Based on the above calculation, the volume change of the micro-cone hole is directly proportional to the driving voltage during a vibration cycle. The volume change caused by the fluctuation of the inner wall of the micro cone hole can provide the driving force for atomization of the atomizer.

## Numerical Simulation of the Formation and Separation Process of Droplets

Actually, during the atomization process, the formation and separation process of droplets belongs to a typical gas-liquid two-phase flow. It brings difficulties in tracing of the free interface of the gas-liquid two-phase flow due to their complexity.

### Establishment of Governing Equations

In this study, the CLSM approach is used to describe the characters of liquid interface. In this method, the 0.5 level is used as the interface of liquid and air. The CLSM can present a high calculation accuracy to distinguish and divide the liquid and air. The mass and momentum transfer of fluid are expressed by the incompressible N-S equation (including surface tension). Because of the velocity of liquid and air is lower than that of sound, they can be treated as incompressible fluid. Accordingly, the N-S equation and continuity equation can be obtained as followings.

12 $$\left\{ {\begin{array}{*{20}l} {\left( {\rho \vec{u}} \right)_{t} + \nabla \cdot \left( {\rho \vec{u}\vec{u}} \right) = - \nabla p + \frac{1}{Re}\nabla \left( {\mu \left( {\nabla \vec{u} + \left( {\nabla \vec{u}} \right)^{T} } \right)} \right) + \frac{\rho }{{Fr^{2} }}\vec{e}_{g} + \frac{1}{We}\vec{F}_{sv} ,} \\ {\nabla \cdot \vec{u} = 0,} \\ {\Phi_{t} + \nabla \cdot (\Phi \vec{u}) = 0,} \\ \end{array} } \right.$$

where $$Re = {\raise0.7ex\hbox{${\rho_{{{\text{ref}}}} u_{{{\text{ref}}}} l_{{{\text{ref}}}} }$} \!\mathord{\left/ {\vphantom {{\rho_{{{\text{ref}}}} u_{{{\text{ref}}}} l_{{{\text{ref}}}} } {\mu_{{{\text{ref}}}} }}}\right.\kern-\nulldelimiterspace} \!\lower0.7ex\hbox{${\mu_{{{\text{ref}}}} }$}}$$, $$Fr = u_{{{\text{ref}}}} .\left( {l_{{{\text{ref}}}} \cdot g} \right)^{{ - \frac{1}{2}}}$$, $$We = \frac{{\rho_{{{\text{ref}}}} u_{{{\text{ref}}}}^{2} l_{{{\text{ref}}}} }}{\sigma }$$. *ρ*_ref_, *u*_ref_, *l*_ref_, *μ*_ref_ are density, viscosity, length, velocity, respectively, they are all constant parameters. $$\sigma$$ is surface tension coefficient.

For CLSM, the 0.5 level is used as the interface of liquid and air, in which the factor of 1 is used for liquid, and the factor of 0 is used for air. At the interface between liquid and air, the parameter of *Φ* transition from 0 to 1 is smooth. Therefore, the convection equation of the level set function after reinitialization can be rewritten as follows:

13 $$\frac{\partial \Phi }{{\partial t}} + \vec{u} \cdot \nabla \Phi + \gamma \left[ \begin{gathered} \left( {\nabla \cdot \left( {\Phi (1 - \Phi )\frac{\nabla \Phi }{{\left| {\nabla \Phi } \right|}}} \right)} \right) - \hfill \\ \varepsilon \nabla \cdot \nabla \Phi \hfill \\ \end{gathered} \right] = 0,$$

where, $\varepsilon$ is determined by the thickness of the interface between liquid and air, $\varepsilon = hc/2$, *hc* represents the size of a typical grid of droplets passing through the region. $\gamma$ is determined by the amount of reinitialization, that is, the stability of the level set function *Φ*, which is mainly affected by the interface velocity of liquid and air.

Surface tension plays a dominant role in the process of droplet spraying. For the simulation, the surface tension should be taken into account to achieve the droplet velocity and size.

The continuous surface tension model is adopted under this condition.

14 $$F_{sv} = \sigma \delta (\Phi )\kappa (\Phi ){\varvec{n}},$$

where ${\varvec{n}}$, $\sigma$, $\kappa (\Phi )$, $\delta (\Phi )$ are direction vector of interface, surface tension coefficient, curvature, Dirac delta function of interface, respectively.

In order to improve the calculation accuracy of surface tension, the surface tension is further modified by the weak solution form of momentum equation (Eq. (15)).

15 $$F_{sv} = \nabla \cdot \left[ {\sigma \left( {{\varvec{I}} - \left( {{\varvec{nn}}^{\text{T}} } \right)} \right)\delta (\Phi )} \right],$$

where ${\varvec{I}}$ is defined as an identity matrix.

In order to simplify the calculation, Dirac delta function is approximated as Eq. (16).

16 $$\delta (\Phi ) = 6\left| {\Phi (1 - \Phi )} \right|\left| {\nabla \Phi } \right|.$$

### Simulation Model

**Figure 7:**
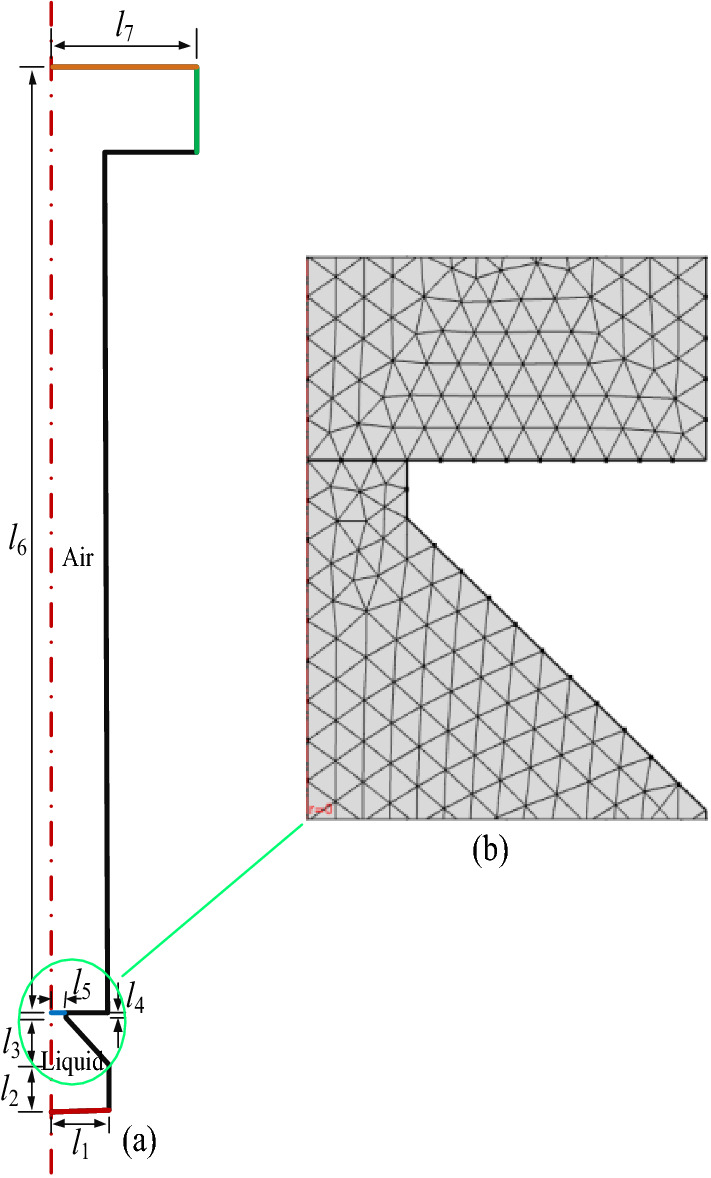
The applied simulation model and mesh partition used in CLSM method

Figure 7(a) shows the geometric outline of the liquid gas interface containing the air and the liquid chamber. In Figure 7(a), the symbols of *l*_1_, *l*_2_, *l*_3_, *l*_4_, *l*_5_, *l*_6_, *l*_7_ and *l*_8_ represent the radius of the liquid storage chamber, the thickness of the liquid storage chamber, the length of the conical nozzle, the length of the tip of the conical nozzle, the radius of the tip of the conical nozzle, the length of the air chamber, and the outlet radius, respectively. The related characteristic dimensions of the geometric contour are listed in Table 1. In order to precisely analyze the cross section between liquid and air, adaptive mesh generation is used, that is, in the simulation process, with the interface moving, the mesh will be updated automatically. Figure 7(b) shows the mesh generation diagram, we using numerous triangular meshes to divide the model, we can obtain 73196 mesh nodes and 721633 elements. Because the model is set as an axisymmetric image and only half one side is taken as a representation for calculation, and then the other one side model can be obtained through the mapping principle.

**Table 1:** Feature size of simulation model

Liquid chamber					Air chamber	
*l*_1_ (mm)	*l*_2_ (mm)	*l*_3_ (mm)	*l*_4_ (mm)	*l*_5_ (mm)	*l*_6_ (mm)	*l*_7_ (mm)
0.08	0.05	0.05	0.01	0.02	1	0.2

In present study, the atomization process of atomizer is also simulated by COMSOL software. The temperature of water is selected as the parameter of liquid part, and the surrounding medium is air. For the corresponding calculation of gas-liquid two-phase flow, the fluid region covers the water and air, and their physical properties are required to be fixed before the calculation. Table 2 shows the physical properties of liquid materials, including the density of liquid materials, the dynamic viscosity of liquid materials, and the surface tension of liquid materials, where the surface tension of air is set to the value of 0.

**Table 2:** Physical properties of liquid materials

Medium	Density (kg/m^3^)	Dynamic viscosity (N·s/m^2^)	Surface tension (N/m)
Water	997.07	8.934 × 10^−4^	0.072
Air	1.225	1.789 × 10^−5^	

In Figure 7(a), the lines marked by the colors of red, green, black and blue denote the inlet, the outlet, the wall, and the interface between gas and liquid, respectively. Additionally, the water liquid below the interface is indicated as blue line, and air is above the interface, and all the wall surfaces are supposed with no sliding. The target (brown part) is set as “wetted wall” condition with a contact angle of π/2. The outlet is set to a constant pressure of 1 atmosphere. By applying an inlet velocity *v*(*r*) as the vibration velocity of the piezoelectric vibrator, the instantaneous velocity in *z* direction is: *v*(*r*,*t*) = sin(*wt*)·*v*(*r*), the unit of *t* is s, *w* is frequency. When a sinusoidal signal is applied, the water fills the entire conical nozzle.

## Results and Discussion

### Process of Droplet Formation

**Figure 8:**
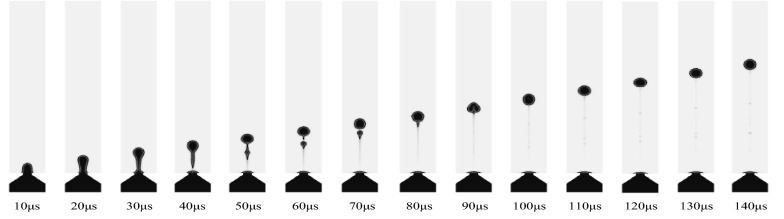
Process of droplet formation

In this study, the research object of the atomized droplet is pure water, and its physical properties are listed in Table 2. The process of droplet formation is simulated, and the changes of velocity and pressure in the process of droplet formation are discussed. In this case, the entrance boundary condition is set as a sine function with the maximum speed of 0.35 m/s, and the droplet spray process is obtained as shown in Figure 8. The process can be divided into several important stages in the droplet spray process: elongation, necking, fracture, formation of satellite droplet, fusion of satellite droplet and main droplet, droplet formation and other stages.

**Figure 9:**
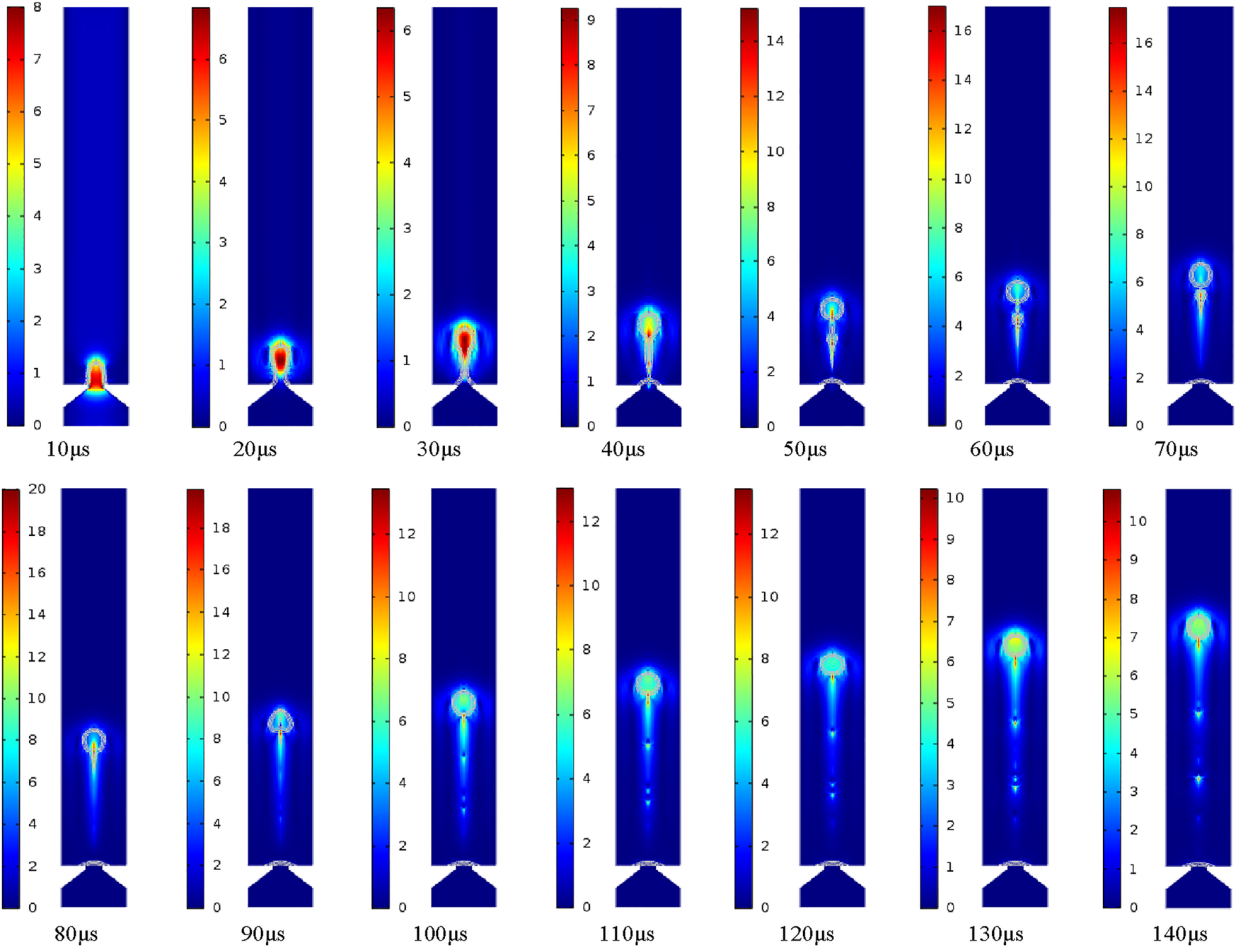
Velocity nephogram of the process of droplet formation

**Figure 10:**
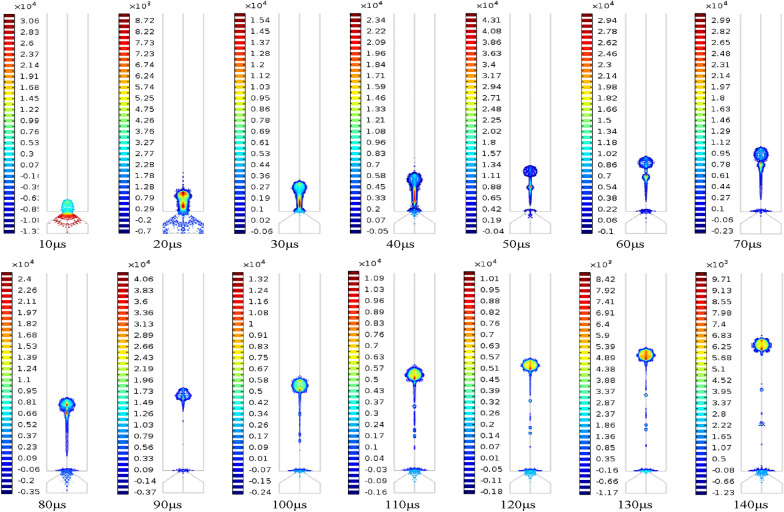
Pressure nephogram of the process of droplet formation

At the initial stage, due to the positive pressure inside the micro cone hole, the liquid will gradually flow out to form a liquid column. After a period of time, a “tongue like” bulge will be produced. Under the action of surface tension, the front end of the liquid column will shrink into a circle. It can be seen from the velocity nephogram of the process of droplet formation shown in Figure 9 that the velocity of the fluid in the central region of the micro cone hole is higher than that of the nearby fluid. It can be explained by the fact that the fluid near the wall of the micro cone hole is much more significantly affected by the wall adhesion force. Furthermore, as the pressure nephogram of the droplet formation process shown in Figure 10, the internal pressure of the droplet is greater than the external pressure, and thus the difference in the pressure can provide the driving force for the movement of the droplet. For the duration of 21 μs, the liquid column is suppressed extending, and then necking phenomenon occurs under the combined action of negative pressure, inertia, pipe wall friction and surface tension in the micro cone hole. At this stage, through the velocity nephogram, it is apparent that the velocity of the front end of the droplet is larger than that of the back end, so that the liquid column will be gradually elongated. The front part and backward part of the liquid column is named as the main droplet and tail droplet, respectively. The velocity difference between the main droplet and the tail droplet will keep the liquid column growth. At 38.8 μs, the liquid column will break completely and spray out. According to the velocity nephogram above, the front-end velocity of droplet is greater than that of back-end droplet. As time prolongs to 52.3 μs, the main droplet and tail droplet will be disconnected to form satellite droplet. Subsequently, the main droplet will form a stable circle under the combined action of the main air force and surface tension. After the formation of the spherical droplet, the air resistance of the droplet is enhanced, and the droplet speed is reduced. Simultaneously, it can be seen from the pressure nephogram that the pressure inside the satellite droplet is greater than that of the main droplet, which can provide more power for the movement of the satellite droplet, so that the satellite droplet will gradually fuse with the main droplet ahead. During the last stage of 71.7 μs, the satellite droplet will fuse with the main droplet.

### Effects of Inlet Velocity on Droplet Formation

**Figure 11:**
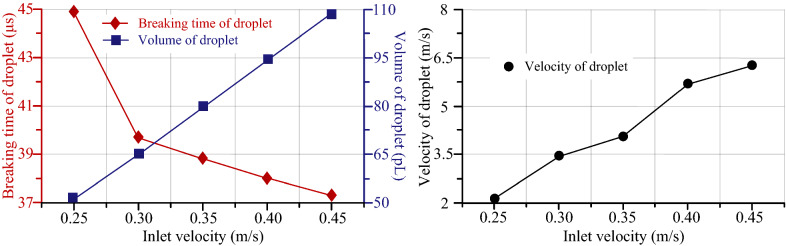
The relationship among breaking time of droplet, volume of droplet, velocity of droplet and inlet velocity

Figure 11 shows the relationships among breaking time of droplet, volume of droplet, velocity of droplet and inlet velocity. The simulation results show that when pure water is selected as the liquid at the temperature of 25 ℃, the volume and velocity of droplet almost linearly increase with inlet velocity increasing, while the breaking time of the droplet decreases. However, when the inlet velocity is greater, this shows that the deformation of the piezoelectric vibrator is larger, and the energy applied to the liquid is also large. With the applied energy increasing, the droplet is easier to get rid of the bondage of the surface tension and spray into the air, so the breaking time of droplet is decreased. Similarly, with the energy increasing, larger initial kinetic energy is achieved for the droplet, and thereby its velocity will be larger. With extent of deformation increasing, the volume of liquid at the injection point will increase in one cycle, so the volume of droplet will be also enhanced.

### Effects of Liquid Temperature on Droplet Formation

**Figure 12:**
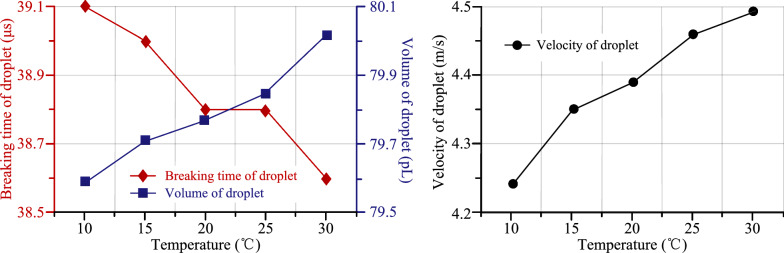
The relationship among breaking time of droplet, volume of droplet, velocity of droplet and liquid temperature

Figure 12 shows the relationships among breaking time of droplet, volume of droplet, velocity of droplet and liquid temperature. The simulation results demonstrate that when the inlet velocity is 0.35 m/s and pure water is chosen as liquid source, the droplet volume decreases and the droplet velocity increases with the temperature and breaking time of liquid increasing. But the overall change trend is not obvious. This phenomenon can be ascribed to the following factors: (1) with the increase of liquid temperature, the viscosity and surface tension of the liquid will decrease. (2) The energy consumed to overcome the viscous resistance will be reduced, and the velocity of the droplet will be increased. (3) With the decrease of viscosity, the resistance of droplet formation will be decreased, and the breaking time of droplet will be decreased, and the volume of droplet will be increased.

### Effect of Liquid Concentration on Droplet Formation

**Figure 13:**
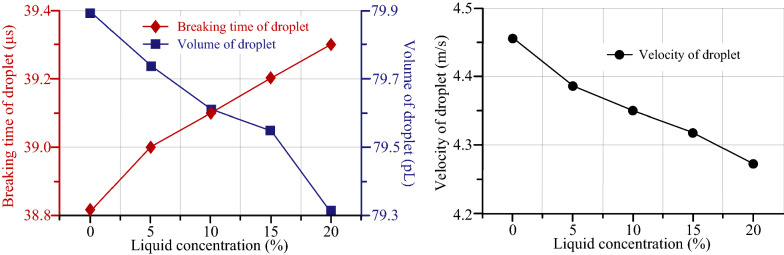
The relationship among breaking time of droplet, volume of droplet, velocity of droplet and liquid concentration

Figure 13 shows the relationship among breaking time of droplet, volume of droplet, velocity of droplet and liquid concentration. It can be concluded from the simulation results that when the inlet velocity is 0.35 m/s and the liquid temperature is 25 ℃, the breaking time and volume of droplet increase, and the velocity of droplet decreases, but the overall change trend is not obvious. The reason for the above phenomenon can be described by the fact that with the increase of the concentration of NaCl solution, the viscosity and surface tension of the liquid increased. The energy is consumed to overcome the viscous resistance increment and the velocity of the droplet reduction. With the droplet viscosity increasing, the resistance of droplet formation is increased, whereas the breaking time of the droplet increases but the volume decreases.

### Effects of Micro-cone Hole Angle on Droplet Formation

**Figure 14:**
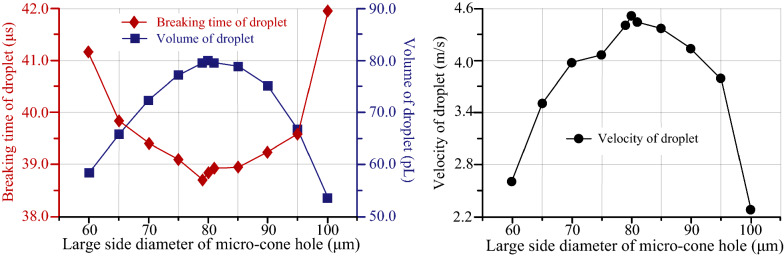
The relationship among breaking time of droplet, volume of droplet, velocity of droplet and large side diameter of micro-cone hole

In order to verify the influences of the cone angle on the droplet formation process, the small side diameter of micro-cone hole is set as 10 μm. By changing the large side diameter of micro-cone hole, the taper hole with different angles can be obtained. Figure 14 shows the relationships among breaking time of droplet, droplet volume, droplet velocity and large side diameter of micro-cone hole. The simulation data show that when the liquid is selected as pure water, the liquid temperature is 25 ℃, the inlet velocity is 0.35 m/s, the breaking time of the droplet exhibits a minimal value of 38.7 μs when the large side diameter of the micro nozzle is 79 μm, the volume of the droplet is 79.8 pL, the velocity of the droplet reach at a maximum value of 4.46 m/s. The reason for the above phenomenon is that the micro-cone hole participates in the formation of droplets. Owing to the variation in the large side diameter of the micro-cone hole, the flow resistance is also changing constantly, which affects the formation of the droplet.

## Conclusions

In this study, the separation and formation process of droplets in a medical piezoelectric atomization device induced by intra-hole fluctuation were investigated. The related atomization mechanisms were revealed from the micro point of view. This work is anticipated to provide theoretical guidance for designing a novel kind of medical piezoelectric atomization device. The conclusions are drawn as follows.

1. In order to tackle the issue that the level set method usually causes mass loss during droplet separation, which affects the accurate positioning of the interface, Conservative Level Set Method (CLSM) is applied to the simulation of the formation and separation of droplets in atomizer. Besides, the droplet separation process of atomizer was simulated by COMSOL software. The simulation model of liquid-gas interface was established, in which the mesh division, material property setting and boundary condition setting were carried out.
2. The simulation results demonstrate that the fluid velocity in the central region of the micro cone hole is greater than that of the nearby fluid, while the internal pressure of the droplet is greater than the external pressure. Such difference in the pressure can provide the driving force for the movement of the droplet. In particular, the effects of inlet velocity, liquid temperature, liquid concentration and the angle of micro-cone hole on the droplet separation process were systematically discussed.
3. Based on the simulation results, it is apparent that the breaking time of droplet decreases with the inlet velocity and liquid temperature increasing, but increases with the concentration of NaCl solution increasing. When the diameter of the large side diameter of the micro-cone hole is 79 μm, the breaking time of droplet is shortened to a value of 38.7 μs. The volume of droplet decrease with the increase of the inlet velocity and the concentration of NaCl solution, whereas increases with the liquid temperature increasing. Under the same conditions, the volume of droplet reaches the largest value of 79.8 pL. The velocity of droplet increases with the inlet velocity and liquid temperature increasing, and decreases with the concentration of NaCl solution increasing, and thus the velocity reaches the maximum value of 4.46 m/s.

## References

[1] Shah B, Modi P, Sagar RS (2020). In silico studies on therapeutic agents for COVID-19: Drug repurposing approach. Life Sciences.

[2] Nioi M, Napoli PE (2021). The waiver of patent protections for COVID-19 vaccines during the ongoing pandemic and the conspiracy theories: lights and shadows of an issue on the ground. Frontiers in Medicine.

[3] Surina S, Martinsone K, Perepjolkina V (2021). Factors related to COVID-19 preventive behaviors: a structural equation model. Frontiers in Psychology.

[4] Dorgham K, Neumann AU, Decavele M (2021). Considering personalized interferon beta therapy for COVID-19. Antimicrobial Agents and Chemotherapy.

[5] Frankel AE, Reddy R, DeSuza KR (2021). Response to pegylated interferon in a COVID-19-positive elderly woman with primary myelofibrosis treated with ruxolitinib. Clinical Cased Reports.

[6] Vecellio L (2006). The mesh nebuliser: a recent technical innovation for aerosol delivery. Breathe.

[7] Lin CY, Meng HC, Fu C (2011). An ultrasonic aerosol therapy nebulizer using electroformed palladium-nickel alloy nozzle plates. Sensors and Actuators A: Physical.

[8] Beck-Broichsitter M, Kleimann P, Gessler T (2012). Nebulization performance of biodegradable sildenafil-loaded nanoparticles using the Aeroneb® Pro: Formulation aspects and nanoparticle stability to nebulization. International Journal of Pharmaceutics.

[9] McCarthy SD, González HE, Higgins BD (2020). Future trends in nebulized therapies for pulmonary disease. Journal of Personalized Medicine.

[10] Pourheidar E, Haghighi M, Kouchek M (2019). Comparison of intravenous ampicillin-sulbactam plus nebulized colistin with intravenous colistin plus nebulized colistin in treatment of ventilator associated pneumonia caused by multi drug resistant acinetobacter baumannii: Randomized open label trial. Iranian Journal of Pharmaceutical Research.

[11] Nguyen AQ, Denault AY, Théoret Y (2020). Inhaled milrinone in cardiac surgical patients: A pilot randomized controlled trial of jet vs. mesh nebulization. Scientific Reports.

[12] Olseni L, Palmer J, Wollmer P (1994). Quantitative evaluation of aerosol deposition pattern in the lung in patients with chronic bronchitis. Physiological Measurement.

[13] J P Mitchell, M W Nagel. Particle size analysis of aerosols from medicinalinhalers, *Powder&Particle*, 2004: 32-65.

[14] Harlow FH, Welch JE (1965). Numerical calculation of time-dependent viscous incompressible flow. Physics of Fluids.

[15] Amsden AA, Harlow FH (1970). A simplified MAC technique for incompressible fluid calculation. Journal of Computational Physics.

[16] Akhlaghi M, Mohammadi V, Nouri NM (2019). Multi-fluid VOF model assessment to simulate the horizontal air-water intermittent flow. Chemical Engineering Research & Design.

[17] Yin Y, Zhu WD, Li JX (2021). Structural topology optimization using a level set method with finite difference updating scheme. Structural and Multidisciplinary Optimization.

[18] Zong HM, Liu H, Ma QP (2019). VCUT level set method for topology optimization of functionally graded cellular structures. Computer Methods in Applied Mechanics and Engineering.

[19] Jiang Y, Li H, Chen C (2018). Calculation and verification of formula for the range of sprinklers based on jet breakup length. International Journal of Agricultural & Biological Engineering.

[20] Olsson E, Kreiss G, Zahedi S (2007). A conservative level set method for two phase flow II. Journal of Computational Physics.

[21] Bahbah C, Khalloufi M, Larcher A (2019). Conservative and adaptive level-set method for the simulation of two-fluid flows. Computers & Fluids.

[22] Antepara O, Balcazar N, Oliva A (2021). Tetrahedral adaptive mesh refinements for two-phase flows using conservative level-set method. International Journal for Numerical Methods in Fluids.

[23] de Luna MQ, Kuzmin D, Kees CE (2019). A monolithic conservative level set method with built-in redistancing. Journal of Computational Physics.

[24] Howard AA, Tartakovsky AM (2021). A conservative level set method for N-phase flows with a free-energy-based surface tension model. Journal of Computational Physics.

[25] Cai YF, Zhang JH, Zhu CL (2016). Theoretical calculations and experimental verification for the pumping effect caused by the dynamic micro-tapered angle. Chinese Journal of Mechanical Engineering.

[26] Yan QF, Sun WT, Zhang JH (2020). Study on the influencing factors of the atomization rate in a piezoceramic vibrating mesh atomizer. Applied Sciences.

[27] Yan QF, Sun WT, Zhang L (2021). Effects of vibration characteristics on the atomization performance in the medical piezoelectric atomization device induced by intra-hole fluctuation. Chinese Journal of Mechanical Engineering.

[28] Yan QF, Wu CY, Zhang JH (1836). Effect of the dynamic cone angle on the atomization performance of a piezoceramic vibrating mesh atomizer. Applied Sciences.

[29] Yan QF, Zhang JH, Huang J (2018). The effect of vibration characteristics on the atomization rate in a micro-tapered aperture atomizer. Sensors.

[30] Zhang JH, Yan QF, Huang J (2018). Experimental verification of the pumping effect caused by the micro-tapered hole in a piezoelectric atomizer. Sensors.

